# Induction of chronic prostatitis does not alter the innate contractile properties of the prostate or urethra in rats

**DOI:** 10.1038/s41598-025-06531-7

**Published:** 2025-06-20

**Authors:** Ozgu Aydogdu, Gwenaelle Black, Patrik Aronsson, Thomas Carlsson, Michael Winder

**Affiliations:** 1 Department of Urology, Institute of Clinical Sciences, Sahlgrenska Academy, University of Gothenburg, Gothenburg, Sweden; 2 Department of Pharmacology, The Sahlgrenska Academy, University of Gothenburg, Medicinaregatan 13, Göteborg, 405 30 Sweden

**Keywords:** Chronic pelvic pain syndrome, Prostatitis, Urethra, Prostate, Organ bath, In vitro, Physiology, Pathogenesis, Urology

## Abstract

The current study aimed to examine how smooth muscle contractile properties and expression of functional proteins in the urethra and prostate are affected in an animal model of chronic prostatitis/chronic pelvic pain syndrome (CPPS). Thirty-two male Sprague-Dawley rats received an intraprostatic injection with saline or zymosan, serving as control and a model for CPPS, respectively. Two weeks later, the urethra and dorsal prostate were excised and studied functionally in organ baths. Following this, protein expression and urethral inflammation was examined immunohistochemically. Neither prostate nor urethra showed any significant changes in contractility compared to the control group, despite a tendency for increased cholinergic contractile responses in the CPPS urethra. Induction of CPPS led to an increased expression of muscarinic M3 receptors in the urethra. In the prostate, there were no significant differences in protein expression. HE staining showed no signs of inflammation in the urethra in either group. Previous studies have shown that CPPS can alter bladder contractility. Currently, CPPS did not affect contractile responses in neither prostate nor urethra. These findings are consistent with the theory of prostate-to-bladder cross-organ sensitization. Future studies exploring this may be of great relevance in the development of new treatment options for CPPS.

## Introduction

Several animal studies have demonstrated that induction of chronic inflammation of the prostate leads to symptoms of bladder overactivity^1–3^. This is in line with clinical findings that consistently demonstrate co-morbidity between chronic prostatitis, also known as chronic pelvic pain syndrome (CPPS), and bladder dysfunction, most commonly overactivity^4^. Management of patients with CPPS is challenging due to heterogeneous symptoms and uncertain diagnostic criteria. In addition, CPPS is probably an underdiagnosed disease due to other similar diseases having overlapping symptoms^1,5^. Since CPPS has a significant negative impact on quality of life, there is a great need for effective treatment options^4^.

A recent animal study has suggested that bladder overactivity that arises as a result of chronic prostatitis can be ameliorated by treatment with a soluble guanylate cyclase (sGC) activator^6^a drug which mimics the in vivo effects of nitric oxide (NO). However, to validate these findings, it is crucial to understand the exact mechanism by which prostate inflammation affects bladder function. It has been suggested that chronic prostatitis can lead to changes in the bladder through cross-organ sensitization^7–9^. Specifically, that inflammation in the prostate affects bladder function by sensitizing, or activating, shared nerves. However, this remains to be proven conclusively. Another possibility is that the induction of bladder overactivity stems from local effects in the prostate, merely disrupting other parts of the lower urinary tract due to proximity. Thus, this scientific question requires further exploration.

Previous animal studies on isolated tissues have revealed that chronic inflammation of the prostate can alter the innate contractile properties of the bladder^10^. This further demonstrates the tentative possibility of cross-organ sensitization between the prostate and bladder. But to be certain that the changes in the bladder are not merely a result of local tissue-to-tissue interaction, the innate contractile properties of other functionally important tissues in the lower urinary tract, i.e. the prostate and the urethra, need to be investigated as well.

**Fig. 1:**
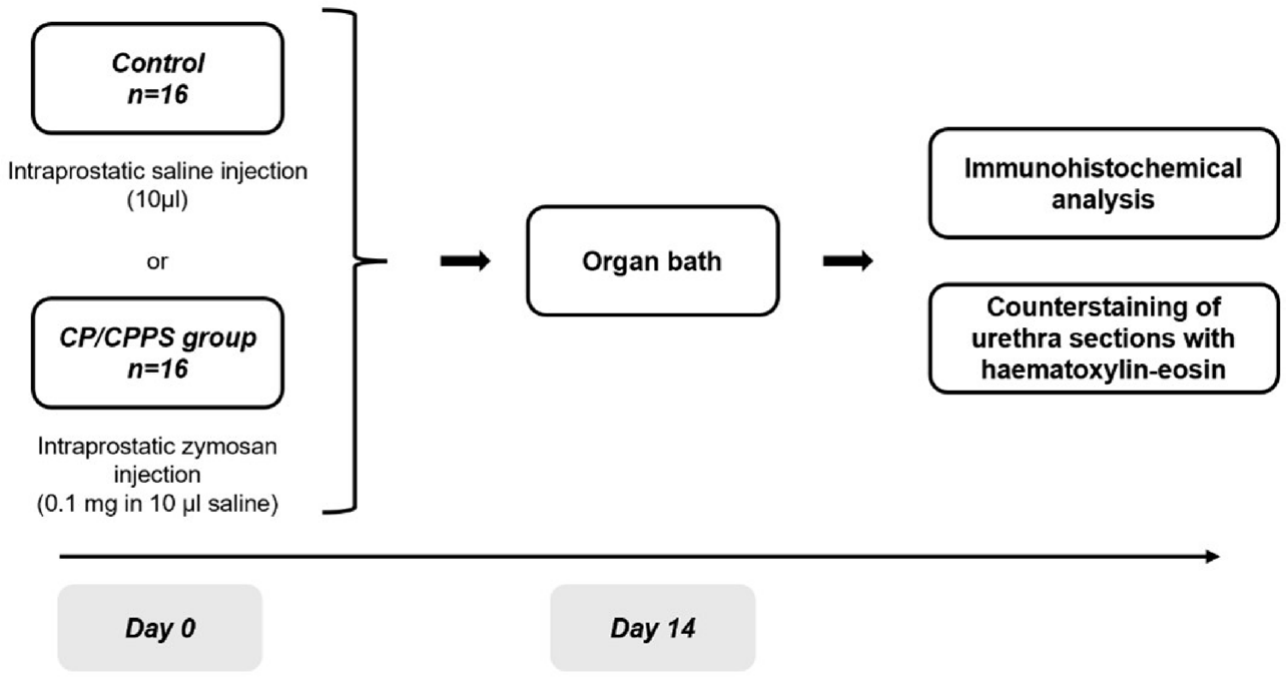
Study outline. Rats were randomly divided into a control group, receiving an intraprostatic saline injection, or a group modelling chronic prostatitis/chronic pelvic pain syndrome (CP/CPPS), which received an intraprostatic zymosan injection. On day 14, the urethra and dorsal prostate were excised and their contractile responses were examined in an organ bath setup. Later, protein expression was analysed by immunohistochemistry, and urethra sections were counterstained with haematoxylin-eosin in order to study inflammatory changes.

**Fig. 2:**
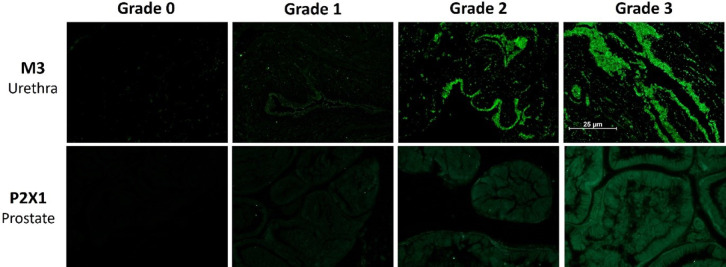
Representative micrographs for grading of protein expression. The grade of receptor expression was scored blindly on a relative scale from 0 to 3 where 0 represented no receptor expression and 3 represented very strong receptor expression. Top row shows representative micrographs for expression of muscarinic M3 receptors in the urethra. Bottom row shows representative micrographs for expression of purinergic P2 × 1 receptors in the prostate.

The objective of the present study was to investigate whether (a) contractile properties and (b) functional receptor and enzyme expression in the prostate and urethra are altered in a state of chronic prostatitis. For this purpose, rats received intraprostatic injections with either zymosan, to chemically induce prostate inflammation, or saline, serving as control. Two weeks later, the animals were euthanized and their prostate and urethra were excised and mounted in organ baths to examine innate contractile responses to electrical field stimulation (EFS), the muscarinic receptor agonist methacholine, the α_1_-adrenoceptor agonist phenylephrine, the P2 purinoceptor agonist ATP and nitric oxide (NO). Subsequently, the tissues were fixed in paraformaldehyde (PFA) and immunohistochemically stained for functional proteins, i.e. muscarinic M3 receptor, α_1_-adrenoceptor, P2 × 1 purinoceptor and soluble guanylate cyclase (sGC) (Figs. 1, 2).

## Results

**Fig. 3:**
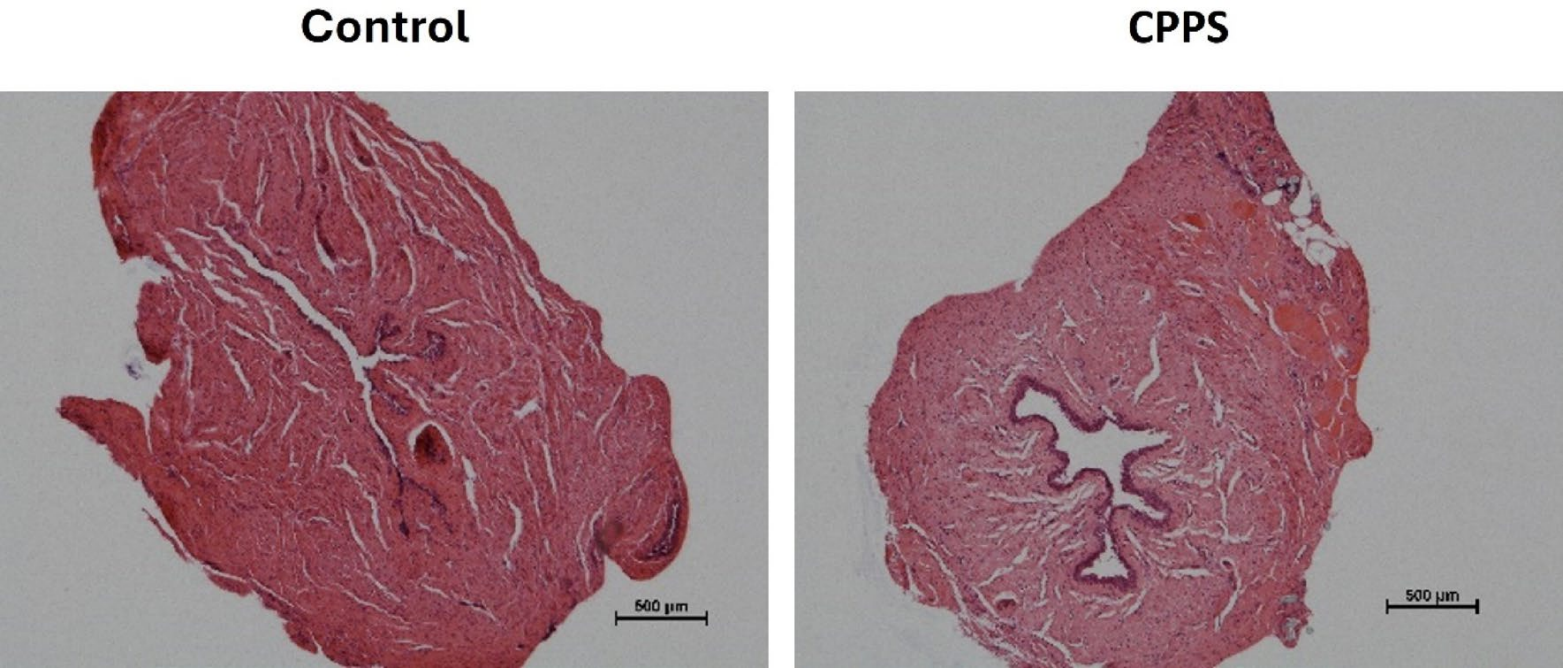
Representative micrographs of haematoxylin-eosin staining to investigate inflammatory changes in the urethra. *CPPS* chronic pelvic pain syndrome.

There were no significant differences in tissue weight between the groups in terms of dorsal prostate (95 ± 7 and 99 ± 11 mg in control and CPPS, respectively) or urethra (19.6 ± 2.3 and 20.1 ± 2.1 mg). While the dorsal prostate tissues in the CPPS group were clearly inflamed, which is in line with what has been demonstrated previously^1^the HE staining of the urethra tissues showed no signs of inflammatory changes in any of the groups (Fig. 3).

### Urethra contractile responses

**Fig. 4:**
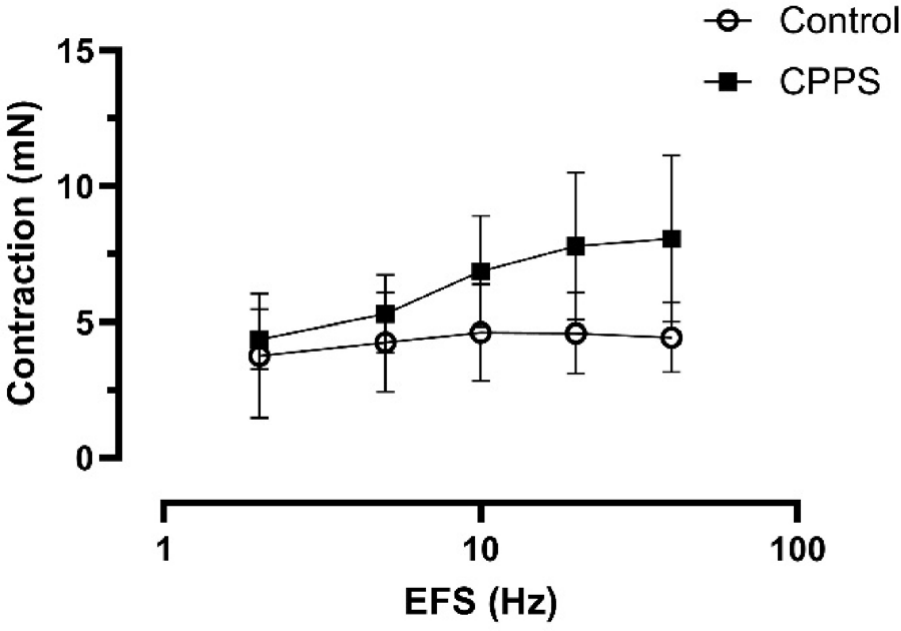
Mean contractile responses of isolated urethra to electric field stimulation (EFS) at 2, 5, 10, 20 and 40 Hz. No significant differences could be detected by two-way ANOVA, even though there is a trend for greater contractions in the CPPS group. The vertical bars indicate SEM. *CPPS* chronic pelvic pain syndrome.

**Fig. 5:**
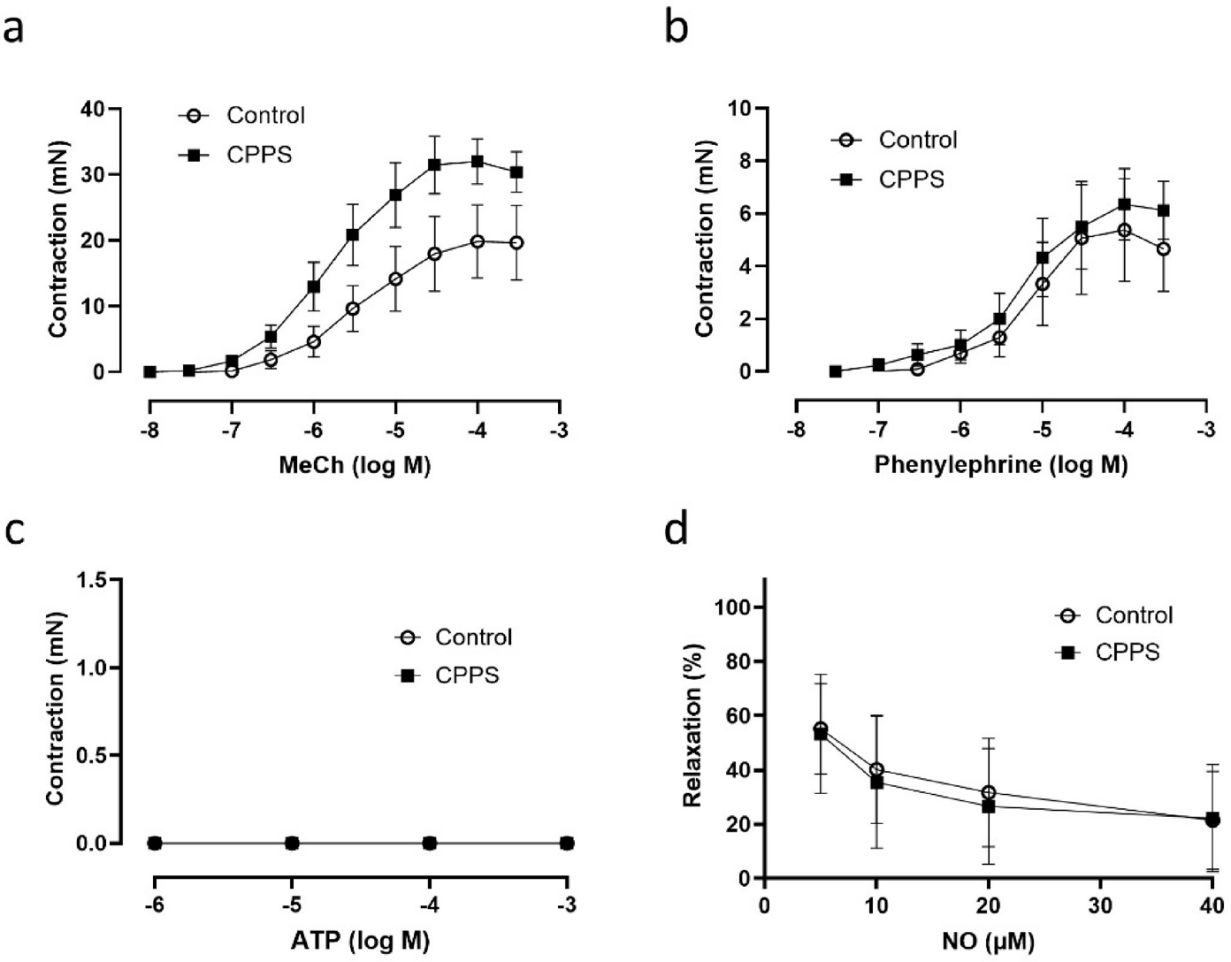
Mean contractile responses of isolated urethra to (**a**) methacholine, (**b**) phenylephrine, (**c**) ATP, and (**d**) relaxatory responses to nitric oxide (NO). No significant differences could be detected by two-way ANOVA, even though there is a trend of attenuated cholinergic contractile responses in the CPPS group. The tissues were unresponsive to ATP. The vertical bars indicate SEM. *CPPS* chronic pelvic pain syndrome.

As compared to controls the contractile responses to EFS were slightly but not statistically significantly higher in the CPPS group (Fig. 4). Although the contractile responses to MeCh were slightly higher in the CPPS group, there were no significant differences as compared to controls (Fig. 5a). No significant differences could be observed between the groups regarding responses to phenylephrine or NO (Fig. 5b, c), and no responses could be observed at all to ATP (Fig. 5d).

### Dorsal prostate contractile responses

**Fig. 6:**
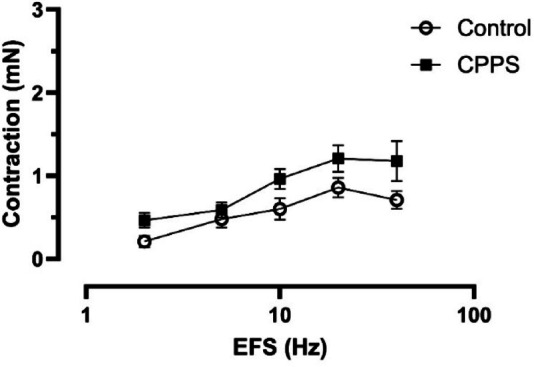
Mean contractile responses of isolated dorsal prostate to electric field stimulation (EFS) at 2, 5, 10, 20 and 40 Hz. No significant differences could be detected between the groups by two-way ANOVA. The vertical bars indicate SEM. *CPPS* chronic pelvic pain syndrome.

**Fig. 7:**
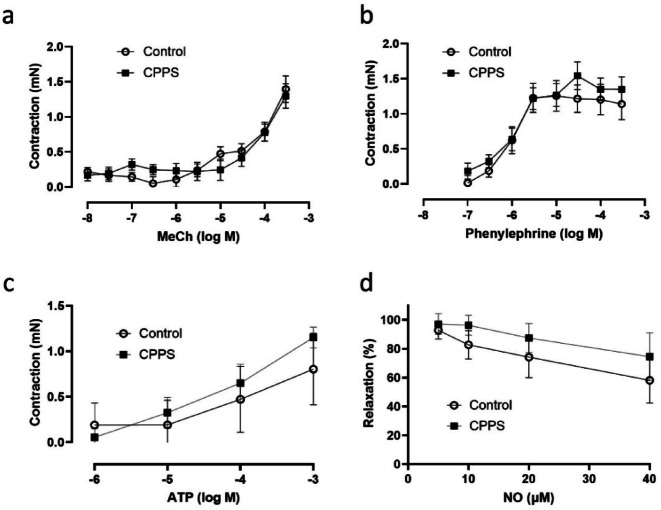
Mean contractile responses of isolated dorsal prostate to (**a**) methacholine, (**b**) phenylephrine, (**c**) ATP, and (**d**) relaxatory responses to nitric oxide (NO). No significant differences could be detected by two-way ANOVA. The vertical bars indicate SEM. *CPPS* chronic pelvic pain syndrome.

Induction of CPPS did not alter the contractile responses to EFS (Fig. 6). There were no significant differences between the two groups in the contractile responses to MeCh (Fig. 7a), phenylephrine (Fig. 7b), NO (Fig. 7c) or ATP (Fig. 7d).

### Urethra protein expression

**Fig. 8:**
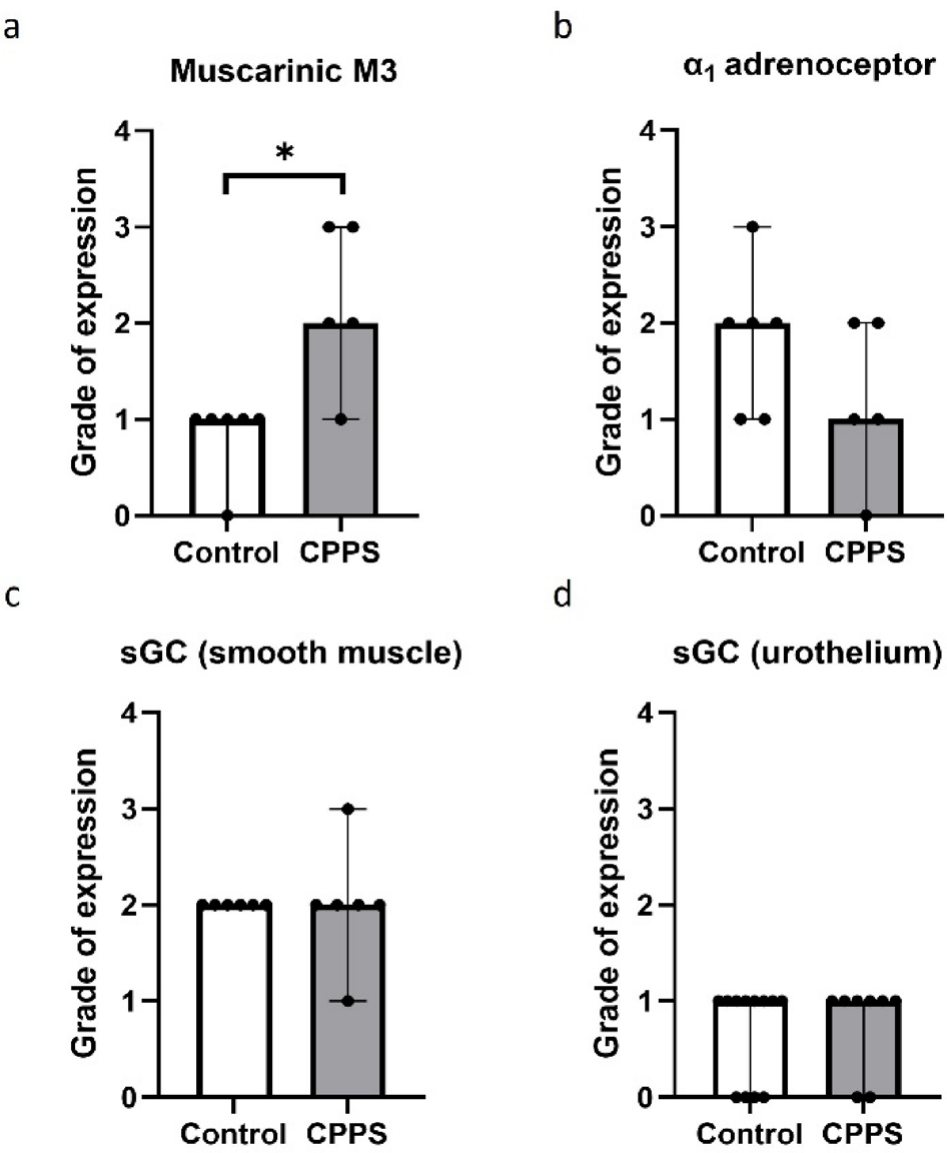
Immunohistopathological analysis of (**a**) muscarinic M3 and (**b**) adrenergic α_1_ receptor expression, and (**c**,**d**) expression of soluble guanylate cyclase (sGC) in the urethra. The vertical bars indicate range. * *p* = 0.013 (Mann-Whitney U test); *CPPS* chronic pelvic pain syndrome.

Muscarinic M3 receptor expression was significantly higher in urethra tissues in the CPPS group, as compared to controls (*p* = 0.013, Fig. 8a). However, there were no significant differences between the groups in terms of adrenergic α_1_ receptor (Fig. 8b) or sGC expression (Fig. 8c, d). No expression of P2 × 1 purinoceptors could be detected in the urethra tissues.

### Prostate protein expression

**Fig. 9:**
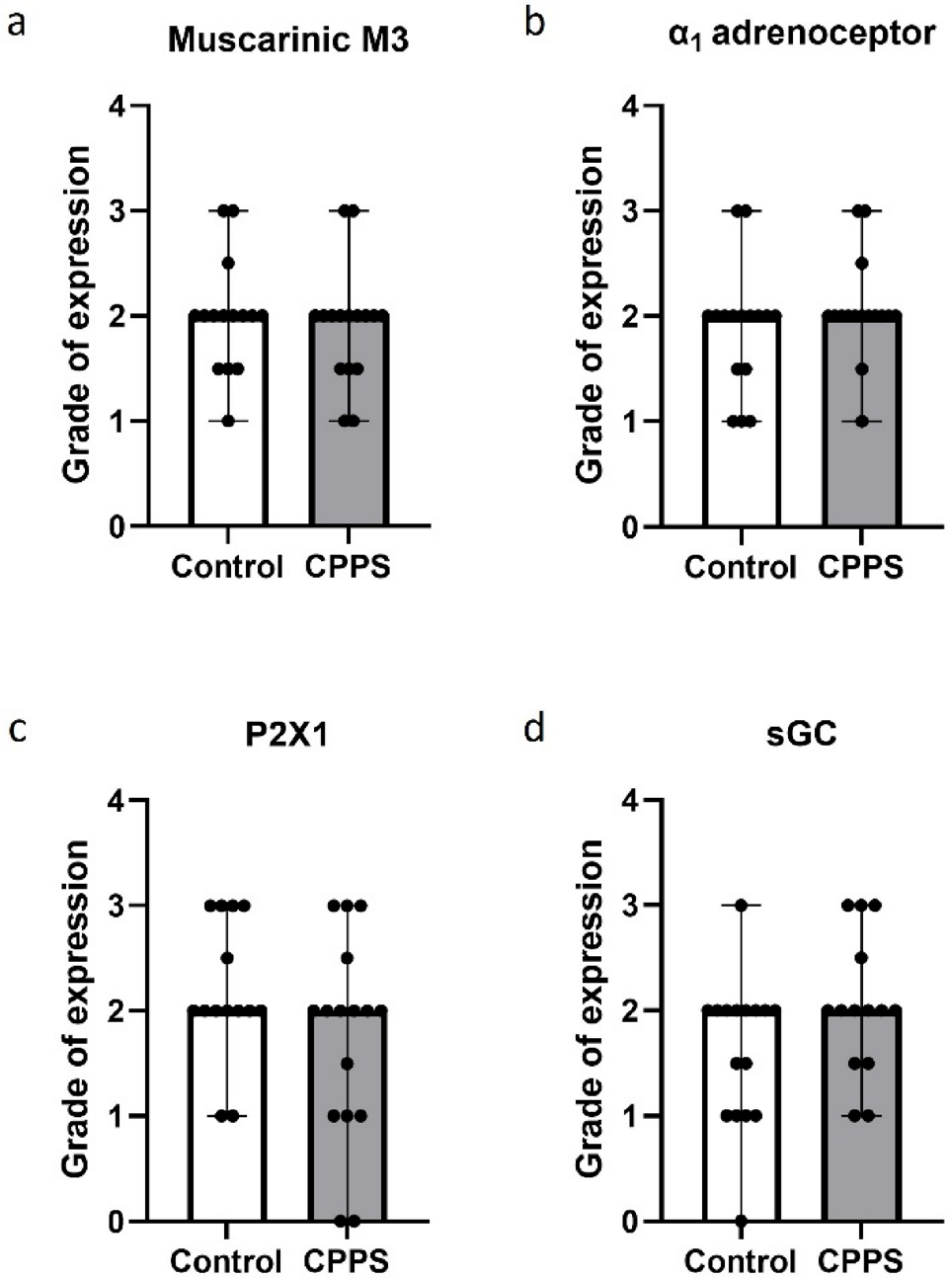
Immunohistopathological analysis of (**a**) muscarinic M3, (**b**) adrenergic α_1_ and (**c**) purinergic P2 × 1 receptor expression, and (**d**) expression of soluble guanylate cyclase (sGC) in the dorsal prostate. The vertical bars indicate range. No significant differences could be detected by Mann-Whitney U test. *CPPS* chronic pelvic pain syndrome.

The immunohistopathological analysis showed no significant differences between the CPPS group and controls in terms of muscarinic M3, adrenergic α_1_, purinergic P2 × 1 or sGC expression in the dorsal prostate (Fig. 9).

## Discussion

The current study aimed to examine if induction of chronic inflammation of the prostate leads to alterations in innate prostate and urethral contractile properties and, further, if the expression of functional receptors and the enzyme sGC is altered. To the best of our knowledge, this is the first study to examine this. Previous studies in rodents in which a similar animal model for CPPS was used showed significant effects on both in vivo and in vitro bladder function, consistently leading to symptoms of bladder overactivity^1–3,6^. This was interpreted as being due to a phenomenon known as cross-organ sensitization. Further, it has been shown that induction of chronic prostatitis leads to alterations in innate bladder contractile properties^10^. Somewhat surprisingly, despite clear signs of inflammation, the dorsal prostate shows neither alterations in innate contractile properties nor in expression of functional receptors or sGC. Similarly, no significant alterations in innate contractile properties could be observed in the adjacent urethra. However, induction of CPPS had a slight, albeit not statistically significant, enhancing effect on the cholinergic contractile responses. Further, the expression of functional muscarinic (i.e. M3) receptors was significantly increased in the urethra in the CPPS group. Therefore, it is not possible to completely rule out any impact of prostate inflammation on urethral contractility. Nevertheless, the current data indicate this impact to be minor, if any.

The responses to EFS were interesting, particularly in the urethra. Contractile properties of the rodent urethra are not very well-studied, in particular longitudinal contraction. In fact, most previous studies have focused on contraction of the internal urethral sphincter, or, as it is termed in rodents which lack an internal urethral sphincter, the proximal urethra^11,12^. The current study is therefore the first to characterize rat urethral longitudinal contractile responses in an organ bath setup. Notably, the EFS responses are significantly lower than the responses to methacholine, possibly indicating release of not only acetylcholine and noradrenaline but also one or several relaxatory signaling molecules such as nitric oxide or adenosine.

The cholinergic contractile responses in the dorsal prostate were minute and unaltered by inflammation. However, the urethral contractile responses to EFS, and even more so the responses to methacholine, demonstrate somewhat augmented cholinergic responses in the urethral tissues from animals with prostate inflammation. The explanation can likely be found when studying the expression of muscarinic M3 receptors, which is increased in the CPPS group. Previous studies have determined the presence of cholinergic nerves and expression of muscarinic receptors throughout the urethra, and urethral cholinergic contractile properties have been demonstrated in several species including humans, pigs and rodents^13,14^. In studies in rodents, activation of urethral cholinergic nerves has been shown to lead to production and co-release of NO, in turn leading to smooth muscle relaxation^15,16^. Thus, with concomitant release of ACh and NO, the effect on the urethra will be partly contractile via ACh, thereby shortening the urethral length, while at the same time exerting a relaxatory effect via NO. One could therefore interpret the contractile cholinergic responses seen in an organ bath as a physiological response that opens the urethra and facilitates the passage of urine. The current study indicates that induction of prostate inflammation further eases the passage of urine, at least initially. However, no changes could be demonstrated regarding nitrergic responses or expression of its target, sGC. One also needs to keep in mind that over time, chronic prostatitis may lead to prostate hypertrophy, which in turn will constrict the urethra and hamper the passage of urine.

The currently observed responses to the α_1_-adrenoceptor agonist phenylephrine were obvious and dose-dependent in both the prostate and urethra, which is in line with previous studies that have examined contractile adrenergic responses in isolated rat tissues^17–20^. The immunohistochemical analysis demonstrated expression of α1 adrenoceptors in both the urethra and the rat dorsal prostate. However, despite induction of inflammation, the expression was not altered. This is interesting when considering that α1-adrenoceptor antagonists are a cornerstone in the treatment of benign prostate hyperplasia (BPH) and that previous studies have shown that expression of α_1_ adrenoceptors is increased in rats with BPH^21,22^. This may therefore highlight a distinct difference regarding adrenergic input between prostate hyperplasia and inflammation. Considering the currently observed responses to phenylephrine and the demonstrated expression of α_1_ adrenoceptors, the present study confirms the ability of rat prostate smooth muscle to contract upon activation of α_1_-adrenoceptors. Previous studies have shown that activation of α_1_-adrenoceptors leads to contraction, while activation of β-adrenoceptors leads to relaxation of urethral circular smooth muscle^17,23^. The currently demonstrated contractile responses to phenylephrine could therefore be interpreted as responses that in vivo may lead to narrowing of the urethra, thereby decreasing the passage of urine. To be noted, the contractile responses of the prostate tissues will, if anything, lead to further narrowing of the urethra. However, contrary to the situation seen during BPH, the current study does not indicate that induction of prostate inflammation alters the adrenergic contractile responses in neither the prostate nor the urethra.

Nitrergic relaxatory pathways have previously been demonstrated in animal studies in both the isolated prostate and urethra^24–26^. Further, previous studies have shown that the main pathway of NO-induced relaxation of smooth muscle is via activation of sGC and the subsequent formation of cGMP^27,28^. Expression of sGC is therefore expected to be a prerequisite for nitrergic relaxatory responses in smooth muscle tissues. The expression of sGC is currently verified, both in the dorsal prostate and urethra. Further, both tissues display dose-dependent relaxatory responses when adding increasing amounts of NO in aqueous solution to the organ baths, even though the importance of nitrergic relaxation seems to be greater in the urethra. Considering the previously shown positive effects of sGC activators on ameliorating bladder overactivity that arises as a result of CPPS^6^the current data show that there may also be additional positive local effects of the drug on both the urethra and prostate. This is in line with other animal studies that have demonstrated positive effects of a sGC activator on bladder outlet obstruction caused by benign prostate hyperplasia^29^.

Purinergic signalling and smooth muscle contractile responses have previously been demonstrated in the rat lower urinary tract^30,31^. Several studies have shown how purinergic signalling plays an increased role in certain disease states^32–34^. Even though it has been hard to demonstrate their significance in the healthy human prostate^35^purinoceptor-induced contractions have been shown to be functionally important in in the rat prostate^36^. Further, previous studies have shown that induction of chronic prostatitis with zymosan leads to a significant increase in the amount of ATP in urine^1^. The current study confirms that functional purinoceptors are present in the rat prostate, showing a dose-response relationship in both the healthy and inflamed prostate. Since the expression of P2 × 1 purinoceptors in the prostate was not altered by induction of inflammation, and the functional responses were similar in both groups, the previously observed increase in ATP production likely originates from the bladder. Meanwhile, no purinergic responses could be observed in the urethra, which is in line with previous studies^33^. Taken together with the observation that no expression of functionally important P2 × 1 purinoceptors could be detected, urethral contraction does not seem to be influenced by activation of purinoceptors.

Decades of studies have concluded that bladder smooth muscle is regulated by several pathways. The main contractile pathway both in humans and rodents is cholinergic^37^but there is significant, mainly relaxatory, input from both adrenergic and nitrergic pathways^38^. Also, in the rodent bladder and in certain disease states in humans, activation of purinoceptors can cause contraction^39^. Currently, it is demonstrated that regulation of prostate smooth muscle seems to be as complex as bladder smooth muscle, with cholinergic, adrenergic, purinergic and nitrergic input. Similarly, regulation of urethral smooth muscle is complex, but possibly without any purinergic influence. However, currently, the urethra showed no apparent signs of inflammation and only expression of P2 × 1 purinoceptors was explored. It therefore remains a possibility that in certain disease states activation of other purinoceptor subtypes, for instance P1 or P2Y receptors, may alter urethral smooth muscle contractility.

There is a potential risk for bladder injury and that zymosan diffuses into the bladder during the induction of prostatitis that requires a surgical procedure. However, this has been previously investigated and the surgical technique used to induce CPPS has been shown to prevent diffusion of zymosan into the bladder^1^. In addition, isoflurane used as the anaesthetic drug during the surgical procedures could also have some effects on the bladder function. However, the data from the groups are comparable since the same anaesthetic drug was used in all groups. Further, choosing to examine longitudinal urethral contraction was a conscious choice. However, future studies should examine the possible effects of induction of prostate inflammation on circular contraction of the urethra. Lastly, examining protein expression after performing an organ bath experiment may affect the examined protein expression. However, all tissues were subjected to the same procedure, rendering them comparable.

## Conclusion

The current study demonstrates the multifaceted regulation of prostate and urethral contractile responses. It is shown that despite induction of chronic inflammation, the contractile responses of the prostate are unaltered. It is further demonstrated that the urethra remains non-inflamed, while a minor increase in cholinergic contractions arises. Thus, the current study strengthens the previously proposed hypothesis that induction of prostate inflammation leads to alterations in bladder function via a complex manner, tentatively by cross-organ sensitization via common nerves. In the future it would be of relevance to explore cross-organ sensitization in appropriate patient populations and in animal models that enable studies of individual nerve activation. If the data from future animal studies verify the current and previous findings and this also holds true for humans, new possibilities would arise for patients in great need of new pharmacological treatment options.

## Methods and materials

Thirty-two adult male Sprague-Dawley rats (350–500 g; Charles River Laboratories, Calco, Italy) were used in the current study. The study was approved by the ethics committee at the University of Gothenburg, Sweden (permit number: 1794/2018). All experiments were performed in accordance with the relevant guidelines and regulations and were designed to minimize the suffering of the animals and were performed in accordance with local guidelines and regulations as well as the ARRIVE guidelines^40^. The number of rats were chosen to ensure obtainment of reliable results, i.e. avoid underpowering, but at the same time minimize the number of the animals used, i.e., avoid overpowering. All drugs were purchased from Sigma Aldrich, St Louis, USA unless otherwise stated.

### Study groups

The surgical procedures were performed as previously described^6^. In brief, the animals were randomly divided into two groups (Fig. 1; *n* = 16 per group). In the first group, vehicle (10 µl sterile saline) was injected directly into the dorsal lobe of the prostate, serving as control. In the second group, rats received an intraprostatic injection with zymosan (0.1 mg in 10 µl sterile saline), to create a functional model for CP/CPPS. Intraprostatic injections were performed with laparotomy under deep anaesthesia using 3% isoflurane (Attene Vet 1000 mg/g, Piramal Healthcare UK Limited, UK) on a thermo-regulated heating pad. After the intraprostatic injection, abdominal muscle and skin were closed with separate surgical sutures. A single dose of buprenorphine (0.1 mg*kg^− 1^ s.c.) was given as postoperative analgesia. On day 14 after the intraprostatic injection with saline or zymosan, the urethra and dorsal prostate were excised under isoflurane anaesthesia and the rats were euthanized using an intraperitoneal overdose of pentobarbitone (> 60 mg/kg; APL, Stockholm, Sweden).

### In vitro organ bath experiments

The excised urethra and dorsal prostate tissues were continuously kept in Krebs solution (CaCl_2_ 1.25 mM, glucose 5.5 mM, KCl 4.6 mM, KH_2_PO_4_ 1.15 mM, MgSO_4_ 1.15 mM, NaCl 118 mM and NaHCO_3_ 25 mM). To measure the innate contractile properties of the tissues, an organ bath setup was utilized. Whole (longitudinal) urethra tissues were used for the organ bath experiments, while the dorsal prostate tissues were divided into two equal pieces. The longitudinal urethra and dorsal prostate tissues were mounted in 20 ml organ baths between a fixed hook and an adjustable steel rod connected to an isometric force transducer (TSD125C, Biopac Systems Inc., Goleta, USA). An MP100WSW data acquisition system and AcqKnowledge software (Biopac Systems) was used to record the contractile responses.

The organ baths were filled with Krebs solution, the temperature was kept at a constant level of 37 °C by a thermo-regulated water circuit and the baths were continuously gassed with 5% CO_2_ in 95% O_2_ to maintain the pH of the Krebs solution at a constant level of 7.4 during the entire experiment. Once mounted, the tissues were pre-stretched to 10 mN, and a stable baseline tension of approximately 5 mN was achieved after 45 min. Both at the beginning and end of each experiment, tissue viability was tested by replacing the Krebs solution in the organ baths with high K^+^ Krebs solution (124 mM; achieved by exchanging Na^+^ with equimolar amounts of K^+^). Only viable tissues were included in the final analysis. All drugs used during the experimental protocol were administered in a volume of 100 µl, thus yielding a dilution of 1:200. The baths were rinsed two times after each added drug and the urethra and dorsal prostate tissues were weighed after the experiments were completed.

### Organ bath experimental protocol

Contractile responses to electrical field stimulation (EFS) were examined by applying a stimulation of 2, 5, 10, 20 and 40 Hz directly into the organ baths at a supramaximal voltage of 50 V and a square wave pulse duration of 0.8 ms for 10–20 s. Contractile responses to the muscarinic agonist methacholine (MeCh) and the α_1_-adrenoceptor agonist phenylephrine were studied by cumulative addition of the drug to the baths at concentrations of 1 × 10^− 8^ M, 3 × 10^− 8^ M, 1 × 10^− 7^ M, 3 × 10^− 7^ M, 1 × 10^− 6^ M, 3 × 10^− 6^ M, 1 × 10^− 5^ M, 3 × 10^− 5^ M, 1 × 10^− 4^ M and 3 × 10^− 4^ M. Contractile responses to the P2 purinoceptor agonist ATP were studied by cumulative addition of the drug at concentrations of 1 × 10^− 6^ M, 1 × 10^− 5^ M, 1 × 10^− 4^ M and 1 × 10^− 3^ M. Relaxatory responses to NO were studied by first inducing contraction with a concentration of MeCh in the EC30-EC50 range (in urethra 1 × 10^− 6^ M and in prostate tissues 1 × 10^− 4^ M), and thereafter cumulatively adding NO in aqueous solution at distinct volumes, which corresponded to NO concentrations of 4 × 10^− 6^ M, 1 × 10^− 5^ M, 2 × 10^− 5^ M and 4 × 10^− 5^ M. The method for how to produce NO in aqueous solution and the calculation of the distinct concentrations of NO has been described previously^27,41^.

### Immunohistochemistry

Following each organ bath experiment, the urethra and dorsal prostate tissues were fixed using PFA (4% in 0.1 M phosphate buffer solution). After fixation for 48 h, the tissues were embedded in paraffin and sectioned into 8 μm thin tissue sections (Histolab Products AB, Gothenburg, Sweden). The expression of muscarinic M3, purinergic P2 × 1 and adrenergic α_1_ receptors, as well as the expression of the enzyme sGC (the main functional target of NO), was examined. The grade of protein expression was scored from 0 to 3 where 0 represented no expression, 1 represented slight expression, 2 represented strong expression and 3 represented very strong expression (Fig. 2). The scoring was performed in a blinded manner. Pictures were taken at 20x magnification and given a random number so evaluation (grading) would be blinded. Four persons each gave an individual grade for each protein expression. An aggregate grade of protein expression for each evaluated tissue was reached by determining the average of each blinded evaluator’s grade. Urethra sections were also counterstained with haematoxylin-eosin (HE) and possible inflammatory changes in the urethra were assessed blindly. A similar counterstaining with HE and subsequent examination of inflammatory changes has been performed previously in dorsal prostate tissues from rats with zymosan-induced prostatitis^1^.

To initiate the immunohistochemical staining procedure, all samples were deparaffinized in xylene (2 × 5 min) and rehydrated gradually for 5 min in 99.5%, 95%, 70% and 50% ethanol. Thereafter, heat-induced epitope retrieval was conducted for 30 min at 70 °C in sodium citrate buffer (pH 5.0; Sigma-Aldrich). Next, the sections were incubated for 10 min in pH 5-adjusted 1 mM copper sulfate in 50 mM ammonium acetate (VWR International, Radnor, PA, USA), to reduce the autofluorescence^42^. Subsequently, to reduce nonspecific background signal, blocking solution containing 1% normal goat serum (NGS; Vector Laboratories, Burlingame, CA, USA), phosphate buffered saline (PBS, pH 7.45; Thermo Fisher Scientific) and 0.1% Triton X-100 (Thermo Fisher Scientific) was used for 10 min.

Primary antibody incubation was carried out overnight in a dark chamber at 4 °C. All primary antibodies were diluted in PBS containing 1% NGS and 0.1% Triton X-100. Incubation without primary antibody was also performed for each staining, as corresponding negative controls. All primary antibodies currently used were polyclonal and raised in rabbit: anti-muscarinic M3 (1:500; AB9018; Sigma-Aldrich), anti-purinergic P2 × 1 (1:1000; p7857; Sigma-Aldrich), anti-adrenergic α_1_ (1:1000; ab3462; Abcam, Cambridge, UK) and anti-sGC antibody (1:100; ab53084; Abcam). Primary antibody incubation was followed by secondary antibody incubation with Alexa Fluor 488 goat anti-rabbit (1:1000, A32731; Thermo Fisher Scientific) at room temperature in PBS containing 1% NGS and 0.25% Triton X-100. All samples were dehydrated gradually in 50%, 70%, 95% and 99.5% ethanol and mounted with Prolong Gold anti-fade reagent with DAPI (P36931; Thermo Fisher Scientific). The samples were stored in dark to prevent loss of fluorescent signals and examined under a Nikon 90i brightfield/fluorescence microscope. Micrographs were recorded utilizing a DS-Fi camera and analyzed with NIS Element 4.40 software (Nikon Corporation, Tokyo, Japan).

### Statistical analysis

Statistical calculations were performed using GraphPad Prism version 9.3.1 (GraphPad Software Inc., San Diego, USA). Two-way ANOVA followed by Bonferroni`s post-hoc test for multiple comparisons was used for statistical comparisons of in vitro organ bath data. Immunohistochemical findings were statistically compared using the Mann-Whitney U test. Statistical significance was regarded for p-values < 0.05. The organ bath data are presented as mean ± SEM, immunohistopathological data are presented as median with range. The data and statistical analysis comply with the recommendations on experimental design and analysis in pharmacology^43^.

## Data Availability

The currently generated and analysed datasets will be made available from the corresponding author upon reasonable request.
